# Twelve tips for rapidly migrating to online learning during the COVID-19 pandemic

**DOI:** 10.15694/mep.2020.000082.1

**Published:** 2020-04-29

**Authors:** John Sandars, Raquel Correia, Mary Dankbaar, Peter de Jong, Poh Sun Goh, Inga Hege, Ken Masters, So-Young Oh, Rakesh Patel, Kalyani Premkumar, Alexandra Webb, Martin Pusic

**Affiliations:** 1 Edge Hill University; 2 SOS Médecins; 3 Erasmus University Medical Center; 4 Leiden University Medical Center; 5 National University of Singapore; 6 University of Augsburg; 7 Sultan Qaboos University; 8 NYU Grossman School of Medicine; 9 University of Nottingham; 10 University of Saskatchewan; 11 Australian National University; 12 Harvard Medical School

**Keywords:** Education, online learning, instructional design, social media, organizational change, computer-assisted learning, remote teaching, COVID-19

## Abstract

This article was migrated. The article was marked as recommended.

The COVID-19 pandemic has resulted in a massive adaptation in health professions education, with a shift from in-person learning activities to a sudden heavy reliance on internet-mediated education. Some health professions schools will have already had considerable educational technology and cultural infrastructure in place, making such a shift more of a different emphasis in provision. For others, this shift will have been a considerable dislocation for both educators and learners in the provision of education.

To aid educators make this shift effectively, this 12 Tips article presents a compendium of key principles and practical recommendations that apply to the modalities that make up online learning. The emphasis is on design features that can be rapidly implemented and optimised for the current pandemic. Where applicable, we have pointed out how these short-term shifts can also be beneficial for the long-term integration of educational technology into the organisations’ infrastructure.

The need for adaptability on the part of educators and learners is an important over-arching theme. By demonstrating these core values of the health professions school in a time of crisis, the manner in which the shift to online learning is carried out sends its own important message to novice health professionals who are in the process of developing their professional identities as learners and as clinicians.

## Introduction

Because of the sudden need for physical distancing during the COVID-19 pandemic, most health professions schools have had to shift a majority of their learning activities online. For some schools, this comes as a shift in emphasis for a curriculum that already has a well-integrated online presence. For others, the shift is challenging in that it requires both cultural and technological adaptations that might otherwise have been planned out over a much longer period, but have been forced to rapidly implement changes to accomplish the educational mission despite the disruption.

Online learning is not one single entity but rather a growing collection of modalities and technologies, which can range from a 160-character Tweet to a fully immersive avatar-based clinical simulation. In this 12 Tips article, we celebrate this diversity while at the same time being fully aware of the current perceived and actual limits of using technology. The adaptability of the professions in the face of an unprecedented pandemic has in many respects been enabled and mediated by our ability to communicate, learn and act through the use of technology.

As members of the AMEE Technology Enhanced Learning (TEL) committee, we present both our collective experience and a survey of the last two decades of relevant published 12 Tips in Medical Teacher and MedEd Publish, as well as our insights on rapidly managing the implementation of educational technology in organisations. We have emphasised the key principles and practical insights for frontline educators to optimise the potential of online learning in their educational environment during this unprecedented global pandemic.

## Organisation Level

### Tip 1: Anticipate and move through the change management stages

In the move to online learning, managing the learners and educators is one of the most challenging and critical aspects. Application of existing evidence-based frameworks and change management models is useful, allowing a shared roadmap for the necessary changes.

**Figure 1.**
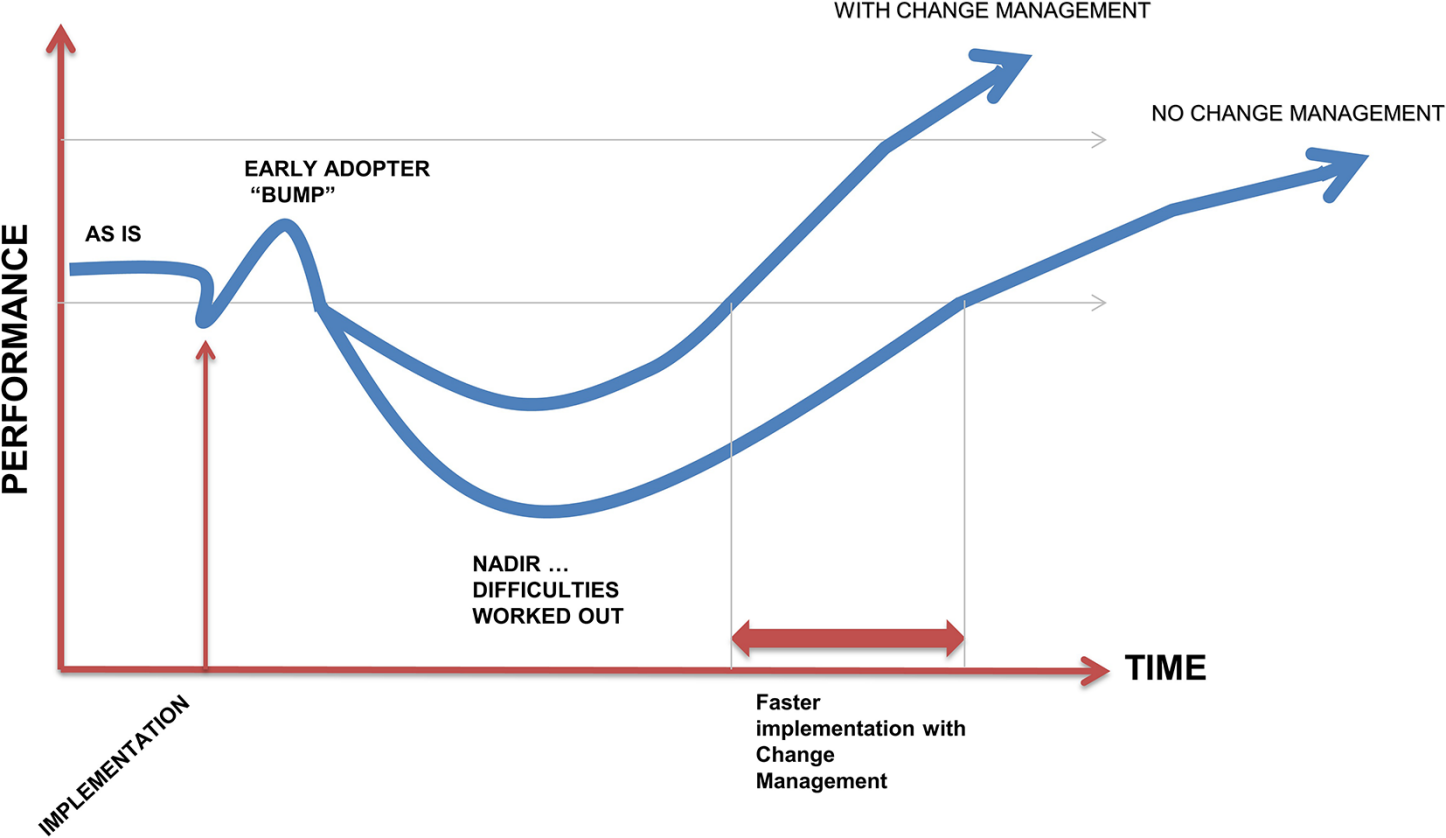
Change management model showing early enthusiasm, followed by the later majority in adapting to the change implementation. (Adapted from Pusic, M.V, Boutis, K, Santen, S. A. and Cuter, W. B. (2019))

The ADKAR organizational change model ( Hiatt, 2006) is focused on the needs of the individuals: Awareness of the need for change (easier in response to the COVID reality); have the Desire to participate in and support the change (such as motivated by self-directed life-long learning); have the Knowledge necessary for change (including where and how to find a suitable technology platform and learning to use appropriate tools for delivery and assessment); Ability to implement required skills and behaviours (including practice with both giving and receiving feedback); Reinforcement to sustain the change (such as program evaluation). The ADKAR model needs to be addressed from the perspective of both learners and educators.As an example, Delgaty (2015) described an action research project where they implemented a distance education module consisting of many of the elements that we will describe in this article, including independent activities, wikis and discussion forums enabling both individual and group tasks. They identified a diverse set of roles that were required for effective implementation, including administrator, manager, content expert and online facilitator.Organisational changes frequently follow a predictable path (see Figure 1). There is a burst of enthusiasm led by early adopters that results in an initial and almost surprising rise in performance followed by a necessary lull in which the rest of the organisation gradually moves into the change. Anticipating these phases and change management can help reduce any problems and informs planning for long term sustainability.

### Tip 2: Use the current Learning Management System

The learning management system (LMS), in most health professions schools, is the platform for communication, content delivery and assessment ( Tibyampansha *et al.*, 2017). It is therefore the fundamental educational technology for structuring the curriculum and conveying constructive alignment of learning outcomes and the educational methods to achieve these outcomes. Given student and staff familiarity with their institutional LMS, basing new online learning activities using a range of modalities, including lectures, tutorials and discussion forums, eases the transition to online learning. However, there are some important elements to consider in ensuring the LMS will aid the learner’s online learning journey when campus closures necessitate learners studying alone at home with no peer or staff face-to-face interaction in physical spaces.

Communication is key in times of uncertainty. Establishing a clear and accessible channel of communication is essential to keep learners and educators regularly informed of the program delivery and to guide them through their learning journey ( Taha *et al.*, 2020). This should include explicit communication about expectations, both what learners should expect during their course of study and the expectations and responsibilities of the learners themselves throughout the course. The LMS analytics can be used to measure and monitor learner achievement of these expectations and identify learners that require additional support. It is important to ensure that the communication is two-way so that learners have diverse avenues to seek advice and guidance.

Considering the learner perspective when organising resources and activities on the LMS is essential, opting for co-creation wherever appropriate. The LMS design should be visually appealing to maximise engagement and intuitive to simplify navigation thus preserving the learner’s cognitive energy for learning. Accessibility should always be considered and, in times of change, it is even more important to provide avenues for evaluation and to rapidly respond to any concerns raised by learners and educators.

## Online Learning Modalities

### Tip 3: Modality - Optimise the potential of online lectures

For the uninitiated, live-streaming lectures can be daunting, but there are a few important recommendations. Speaking to a blank wall can initially appear strange to the presenter but from our experience educators can rapidly adapt and become confident online presenters.

If possible, a system that is integrated into the Learning Management System (LMS) should be used. This will ensure that only registered learners and educators are in the lecture and will avoid having “trolls” and other disrupters. Also, the lecture can be recorded and uploaded into the LMS for later viewing by learners who were unable to attend the lecture or had internet or other problems.

Ensuring that the microphones of all participants are in the mute mode during the presentation will avoid unintended background noise from the learner’s homes. Some lecturers like to keep all cameras on so that they can keep an eye on the engagement of their students. Others prefer to mute all cameras which can also help if there are internet bandwidth issues.

It is important to break the lecture into a series of components, interspersed with small learning activities. This is already a recommendation for face-to-face lectures but is all the more important in online lectures. If the LMS has an audience-response component, this can be used to increase learner engagement in the activities and to maximise the learning opportunity. If not, as the presenter goes through the lecture, a pause can be introduced during which the learners are given a question to answer, which is based on material that has just been presented. This builds in active learning and improves engagement.

If possible, a flipped classroom approach should be used with learning activities, such as reading a book chapter or journal articles or watching a video, before the online lecture and with interactive learner activities and opportunities to ask questions about the pre-learning during the lecture.

### Tip 4: Modality - Optimise online small groups using intentional design

The facilitation of online groups optimises the learning potential of small groups and is similar to the approach used with face-to-face small groups. The two essential aspects of all group facilitation are ensuring that there is overall adherence to a clear objective and supporting the needs of all group members, such as providing opportunities for all members to make a valued contribution.

However, the difficulties that can be experienced when facilitating face-to-face groups are often increased with online groups, especially when the groups are asynchronous (in which participants are only contributing by written messages and at different times, such as by email or most social media) ( Sandars, 2006). There is a lack of non-verbal communication in asynchronous discussions, even with the use of Emojis (‘smiley faces’ and ‘thumbs up’ symbols), and this can lead to a lack of trust between the participants, with the inevitable reduced contribution to the discussion. Synchronous online discussions that enable real-time participation are to be preferred and there are numerous online platforms, such as Skype ( https://www.skype.com/), TopHat ( https://tophat.com/) and Zoom ( https://zoom.us/).

Online groups can be combined with other information resources, such as PowerPoint presentations or videos, to produce a flipped classroom, which can enhance the learning experience. The integration of online group discussions with digital storytelling can also provide powerful reflective learning experiences.

### Tip 5: Modality - Optimise the potential of asynchronous online tutorials

Online tutorials provide learners with a distinct collection of online multimedia learning activities, including the use of video, animations, text based materials and quizzes, which are structured around a patient case presentation ( Yavner, Pusic, Kalet, *et al*., 2015). There are several advantages compared with recorded lectures and other asynchronous resources. The use of multimedia ensures more efficient gains in knowledge and skills since information can be presented using combinations of several different media. Learners become self-directed, with increased motivation and engagement, since they can select online tutorials from a range of different online tutorials that meet their own learning goals at a time and place that fits in with their other demands.

An important decision to be made is whether to build a customised online tutorial or to benefit from the use of high quality available resources, such as Aquifer ( https://aquifer.org/), FOAMed ( https://emergencymedicinecases.com/), MedEdPortal ( https://www.mededportal.org/), the New England Journal of Medicine video series ( https://www.nejm.org/multimedia) or OpenPediatrics ( https://www.openpediatrics.org/). It is most likely that a mixed approach will be required since the available resources may not be appropriate. The educator has an essential role in implementing learning needs assessments to ensure that all online tutorials are aligned to the intended learning outcomes of the curriculum and also curating the available resources, with reviewing to ensure that the content is appropriate and accessible. An iterative evaluation process, including the use of learning analytics, can ensure that the online tutorials are achieving their intended benefits.

Educators can provide a more meaningful online blended flipped classroom learning experience by integrating online tutorials before or after online synchronous discussions. For example, a clinical case discussion can be augmented by online tutorials on the differential diagnosis of clinical presentations or clinical management guidelines.

### Tip 6: Modality - Optimise the potential of online videos

Thanks to the ubiquity of video and audio technology, an iterative customised video development process can be undertaken using commonly available technology, which is informed by educator insight into the needs of the specific audience and underpinned by a knowledge of pedagogy and an understanding of available technology (Dong and Goh, 2016; Norman, 2017). This iterative development process however takes time, effort, attention to detail and resources; all of these aspects are likely to be compromised because of the rapid need to respond during the COVID-19 pandemic.

Optimisation of the use of online videos include a focus on curating open access resources by directing the learners to relevant segments of the videos, encouraging individual reflection and group discussion of the video content, and demonstrating essential procedural and communication skills with peer and educator feedback ( Goh and Sandars, 2019). The concept of blended learning can now be expanded, combining asynchronous self-study of online videos with synchronous live online small group meetings ( Shank, 2020).

Innovative use of real-time video to provide access to patient encounters (with appropriate consent and safety guidelines), as well as to document and provide feedback for practice and delivery of clinical skills, can take place with the ubiquitous availability of mobile devices ( Goh and Sandars, 2020).

### Tip 7: Modality - Optimise the potential of social media

Social media is a widely available technology and offers the possibility of reaching every participant who can offer a variety of perspectives, from other learners, junior doctors in training, healthcare professionals and even patients, that can enrich the learning experience ( Shah, 2017). Twitter journal clubs (Duig, 2016; Topf, 2017; Bolderston, 2018), medical blogging ( Carley, 2017) and collaborative learning platforms ( Haynes, 2014) have been clearly demonstrated to enhance continuing professional development and changing educational practice. However, not everything in a medical curriculum is transferable to an online approach, therefore it is essential for the educator to bear in mind that content alone will not magically translate into an educational experience for the learner.

Content creation and publication on multiple platforms requires a substantial time commitment; so much so that social media management has become a career in its own right. Fostering the social aspect of these platforms by commenting, offering feedback and emulating the community feel are key in engaging learners and improving their learning. In a time of rapid transition to online, identifying early adopters who already have a built infrastructure and online following can help to develop the potential of social media.

The use of content sharing tools, such as Hootsuite ( https://hootsuite.com/) or Buffer ( https://buffer.com/), will save precious time for educators and learners to schedule and automatically post in every chosen platform and an appealing and uniform design for different channels can be created using Canva ( https://www.canva.com/). Another tip is to direct learners to communication and collaboration platforms that have been suggested as effective learning tools ( Coleman, 2019), such as WhatsApp ( https://www.whatsapp.com/) or Slack ( https://slack.com/), where they can socialise, interact with peers and receive feedback. Optimal use of these tools is dependent on user training; all of them offer webinars and quick courses.

### Tip 8: Modality - Optimise the potential of online reflection

Reflection begins with a moment of surprise in the learner and this can be provoked by several factors, from lack of knowledge and skills in the learner to witnessing events that challenge the learner’s view of themselves, others and the wider world. The learner makes sense of the surprise and then takes an appropriate response to resolve the challenge, such as seeking new knowledge or reading further about different world views. The process of reflection is enhanced by having a supportive facilitator and by sharing their experience with others, including learners, clinicians, allied healthcare professionals, patients and care-givers.

Digital storytelling can be a powerful approach for developing the essential reflection component of medical education ( Sandars, Murray and Pellow, 2008). Learners can easily upload a case, which comprises of one or more visual images, to online platforms (such as a discussion forum) and talk through their moment of surprise and how it challenged their views. This can be shared with others and a deep transformative learning experience can be produced that is both individual to the learner but also collective for the participants.

The choice of images about the case is important. It is essential not to compromise the identity of patients or colleagues. Photographs or short videos can be easily captured using mobile devices. However, we recommend that these images should resonate with the learner’s experience. Anonymity can be maintained by using general images, such as a view of a neighbourhood or the direction signs to a ward. There are also a wide variety of images available on the internet but it is important to ensure that they are either in the public domain or have a Creative Commons license, which will require acknowledgment.

## General Online Tips

### Tip 9: If you can’t teach the whole task, start with part-task online training

**Figure 2.**
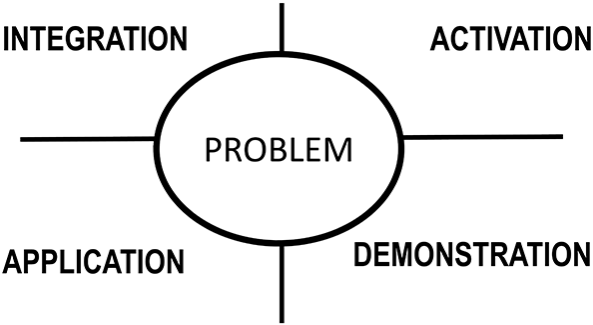
Phases for effective instruction (after Merrill, D.M (2002))

An important part of the health professions curriculum consists of skills training. And while on first sight one might think it is impossible to teach skills online, this is only partially true. Cognitive skills such as clinical reasoning, procedural or academic skills, can be effectively taught online. To design effective online skills trainin, learning is promoted when learners are engaged in solving real-world *problems* (see Figure 2) *.* The following are crucial instructional design principles ( Merrill, 2002): a) *activation* to recall and demonstrate relevant prior knowledge; b) explanation and *demonstration* of tasks and skills (often by the teacher); c) *application* of knowledge or skill in a progression of new tasks receiving feedback (by the learner); and d) *integration* of the newly learned skills in practice (by the learner). These principles can be implemented online in quizzes (activating prior knowledge), tutorials and video’s (explanation and demonstration of skills) and assignments or simulations (applying newly learned skills).

Obviously, practical skills training like the physical examination or laboratory experiments cannot be taught completely online, as a physical presence of a simulation patient or experimental setup is required. However, the first two principles of activation and explanation can still be implemented as online part-task training until the moment that working on-site is possible again. Part-task practice can also prepare learners optimally for the remaining hands-on training component when on-campus education is resumed.

An illustrative example of the part-task approach is learning the interpretation of heart sounds. There are numerous libraries and quizzes related to heart sound interpretation that can be accessed online and used for part-task practice to build a mental model of recognising heart sounds through activation, demonstration and even application of the skill. The learner will then be ready when the time comes to integrate the skill of recognizing heart sounds into clinical learning during hospital placements.

### Tip 10: Simplify the massive online world for learners

Worldwide, large numbers of free massive open online courses (MOOCs) are available in the field of the health sciences. These courses offer educators a great opportunity to replace some of the regular face-to-face teaching, which now is suddenly not feasible anymore, with high-quality materials offered by other institutions. MOOCs offer content in many different formats, such as short video lectures, readings, interactive assignments, tests, quizzes, and discussion forums. In general, learners indicate high satisfaction with the high-quality online materials provided. However wonderful this all appears, the massive online world can be very confusing and overwhelming to learners, especially if the content is not presented in their native language. First, a course needs to be selected that addresses the topics for study at the appropriate level. An important aspect is to find out which dates the course will be available and then send the learners clear instructions on how to enroll into the course. And finally, it is essential to provide learners with clear instructions on how to use the course, which chapters and which assignments to study and which homework they should submit to the educator.

### Tip 11: Encourage and support co-creation of online resources and activities

Co-creation occurs when learners and educators work collaboratively with one another to create online resources and activities ( Bovill *et al*., 2016). There are many interdependent benefits to co-creation besides the development of new or improved online materials and experiences. These can include increased learner engagement and increased self-efficacy, and experiential learning for the development of our future medical educators ( Mercer-Mapstone *et al*., 2017).

It is important to first establish a team with the required skillsets to provide content, curriculum and educational design input for effective collaboration and achievement of the project outcomes. When scoping the project, key recommendations include identification of roles and responsibilities, setting clear deadlines and clearly communicating objectives and expectations. As part of the scoping process it is important to determine what training, support and resources the team requires to deliver the project. This should always include guidance regarding pedagogy, accessibility and copyright. Furthermore, it is vital to ensure that the resources and activities can be made available to learners via institutional online platforms. An important consideration is also the recognition of the contribution of the learners, such as providing academic credits or certificates of extra-curricula activities.

Incorporating peer review and testing into the project management, prior to implementation, for quality control will help to identify and resolve any errors or problems. If the resource is of suitable quality and does not breach copyright, making it available as an open educational resource (OER) to benefit learners beyond the institution should be considered. Evaluation of both the resource and its use by learners (such as by learning analytics) is important for reporting, reflection and scholarship.

### Tip 12: Demonstrate the value for active clinicians of the shift to online learning

The COVID epidemic has required every clinician to learn about the virus and its implications at a rapid rate over a short period of time. Online learning has been an important component of this professional learning.

The global pandemic is also a unique opportunity to observe the way clinicians rapidly acquire new knowledge in active practice. Covid-19 as a disease did not exist previously so clinicians have been addressing the challenge of sharing professional knowledge at what appears to be faster than the spread of disease itself and the predominant methods of sharing this knowledge is mediated through the internet. The number of publications in the World Health Organisation’s online database is rapidly expanding and at the time of writing stands at almost 9000 ( World Health Organisation, 2020). Certain technologies, such as Twitter ( https://twitter.com/explore), amplify the volume and velocity of sharing globally, whilst also providing the platform upon which that knowledge is constructed through continued discourse and debate between the participants ( Carley, 2020). More local knowledge also needs to be developed as the pandemic spreads across the globe causing different patterns of disease depending on the variant of virus mutation affecting a particular country, and the unique local circumstances. Again, technology has been critical in sharing of experience between local experts, as well as devising care pathways, protocols and local policy for the care of patients across different hospital or healthcare settings. Various video-conferencing platforms have also played an important role in fostering a sense of community, as well as promoting a sense of solidarity in demonstrating the indirect social impact of such technologies as well.

We recommend that educators encourage learners to observe and engage in these online learning opportunities since it not only develops their future professional knowledge but also provides an essential opportunity to observe and develop their future approach to continuing professional development.

## Conclusion

The COVID-19 pandemic has resulted in a sudden heavy reliance on the use of online learning. Enhancing teaching and learning during the pandemic requires careful attention to ensure that there is optimization of the range of currently available resources. The process of optimisation requires working with existing organisational cultures to achieve effective change, to adapt the available technologies and to ensure the collaborative participation of educators and learners throughout the process. An important consideration, which may be overlooked whilst heavily engaged in the present situation, is how it will be possible to sustain the use of online learning after the pandemic, instead of simply falling back into a traditional face-to-face teaching routine. Evaluation is essential for sustainability since it provides insights about the extent to which new approaches are achieving their intended and potential benefits, and also the variety of factors that enable and constrain effective online learning. Now is the time to begin the process of evaluating the successful innovative online approaches, as well as the not so successful ones, to iteratively develop and implement online learning that can result in greater and more effective application to health professions education after the pandemic compared with before the pandemic.

## Take Home Messages

• Use the principles of organisational change to anticipate the course of adaptation to online learning
• Good instructional design guidelines are available for all online modalities
• Implementation of online learning is part development and part curation of existing resources
• Blended learning including asynchronous self-study with synchronous live online group discussions can be rapidly implemented
• Encourage co-creation of resources by learners and educators
• Model the adaptability of health professionals to new learning approaches
• Plan for sustainability after the COVID-19 pandemic

## Notes On Contributors

John Sandars, MBChB, MSc, MD, MRCP, MRCGP, FAcadMEd, FHEA is Professor of Medical Education and Director of Medical Education Innovation and Scholarship in the Health Research Institute, Faculty of Health, Social Care & Medicine, Edge Hill University, Ormskirk, UK. ORCID iD: https://orcid.org/0000-0003-3930-387X

Raquel Pereira Correia, MD, is a General Practitioner at SOS Médecins, Paris, France and a Registered Tutor, PgDip in Medical Education, Faculty of Medicine, University of South Wales, Pontypridd, UK. ORCID iD: https://orcid.org/0000-0003-2533-8529

Mary E. W. Dankbaar, PhD, is Team Manager Digital Learning and Innovation and Assistant Professor in Research in Medical Education at Erasmus University Medical Center in Rotterdam, the Netherlands. ORCID iD: https://orcid.org/0000-0003-0880-505X

Peter G. M. de Jong, PhD, AFAMEE, is a Senior Adviser and Assistant Professor for Technology Enhanced Learning at the Center for Innovation in Medical Education at Leiden University Medical Center in Leiden, The Netherlands. ORCID iD: https://orcid.org/0000-0001-9038-3137

Poh Sun Goh, MBBS, FRCR, FAMS, MHPE, FAMEE, is an Associate Professor and Senior Consultant Radiologist at the Yong Loo Lin School of Medicine, National University of Singapore, and National University Hospital, Singapore. ORCID iD: https://orcid.org/0000-0002-1531-2053

Inga Hege, MD, MCompSc is an Associate Professor for Medical Education at the Medical School, University of Augsburg, Germany. ORCID iD: https://orcid.org/0000-0003-4335-5162

Ken Masters, PhD, FDE is Associate Professor of Medical Informatics in the College of Medicine & Health Sciences, Sultan Qaboos University, Oman. ORCID iD: https://orcid.org/0000-0003-3425-5020

So-Young Oh is Program Manager for Digital Learning and Instructional Design at Institute for Innovations in Medical Education (IIME), NYU Grossman School of Medicine, NYULH, New York, NY, USA. ORCID iD: https://orcid.org/0000-0002-4640-3695

Rakesh Patel, MBChB, MRCP, MMed, MD, SFHEA is Clinical Associate Professor in Medical Education, School of Medicine, University of Nottingham, Nottingham, UK. ORCID iD: https://orcid.org/0000-0002-5770-328X

Kalyani Premkumar, MBBS, MD, MSc(Med Ed), PhD, MBA, is a Professor at the College of Medicine, University of Saskatchewan, Saskatoon, SK, Canada. ORCID iD: https://orcid.org/0000-0002-6127-7498

Alexandra L. Webb, BSc, MChiro, PhD, SFHEA, is an Associate Professor at the Medical School, Australian National University, Canberra, Australia where she leads the TEL team. ORCID iD: https://orcid.org/0000-0002-5571-5754

Martin Pusic, MD PhD, is a member of the faculty of Harvard Medical School and the Boston Children’s Hospital, Boston, USA. ORCID iD: https://orcid.org/0000-0001-5236-6598

## Declarations

Ken Masters is an Associate Editor of MedEdPublish, but this article has gone through the normal publishing procedures. Other than that, there are no conflicts of interest.Martin Pusic has been funded for research by Aquifer Inc and the American Medical Association.
