# Supplementary material for: Twelve tips for rapidly migrating to online learning during the COVID-19 pandemic
Source: MedEdPublish (2016). 2020 Apr 29;9:82. [Version 1] doi: 10.15694/mep.2020.000082.1 (PMC10697562; doi:10.15694/mep.2020.000082.1)
Supplement: Peer review history

# Reviewer report: Reviewer response for version 1

**DOI:** 10.21956/mep.20067.r30820

**Published:** 2020-07-15

**Authors:** Viktor Riklefs

**Affiliations:** 1 Karaganda Medical University

This review has been migrated. The reviewer awarded 5 stars out of 5 Very useful recommendations and very nice summary on how to not only rapidly move to online learning, but also on how to develop it. In our own institution, we actually followed through the first six steps intuitively and thought that we are rather smart. But I really liked the last six which are very creative, and we could hardly think of implementing them on our own. I guess AMEE has a very good reason to have the format of exactly 12 tips, because you have to use your imagination to suggest that many tips.

Reviewer Expertise:

NA

No decision status is available

# Reviewer report: Reviewer response for version 1

**DOI:** 10.21956/mep.20067.r30821

**Published:** 2020-05-03

**Authors:** Michael SH Wan

**Affiliations:** 1 University of Notre Dame

This review has been migrated. The reviewer awarded 4 stars out of 5 A very useful summary for medical educators to develop and strengthen the delivery of the curriculum and teaching activities online. The recommended steps are very relevant to the current pandemic environment. The sharing of recourses among schools and the use of online video or materials can significantly reduce the workload of developing new resources.Near peer teaching with senior medical students contributing to the development of online teaching resources and facilitating the delivery could also be considered.

Reviewer Expertise:

NA

No decision status is available

# Reviewer report: Reviewer response for version 1

**DOI:** 10.21956/mep.20067.r30822

**Published:** 2020-04-30

**Authors:** Pat Lilley

**Affiliations:** 1 AMEE

This review has been migrated. The reviewer awarded 5 stars out of 5 This is a well-written paper containing a compilation of useful tips for those delivering online learning. Whilst the stimulus for the paper was the sudden impact of Covid-19 on teaching and learning, the tips represent good practice for online teaching and learning at any time. What comes across is the feeling that, far from just making the best of the situation, online teaching and learning has many advantages if delivered appropriately. The authors identify a range of teaching modalities and suggest that with appropriate course design, a wide range of outcomes can be taught online, including cognitive skills, and to a certain extent practical skills. They also highlight the wealth of existing online resources that can be used for teaching, with appropriate curation of content and guidance for the learner, and also the opportunities for co-creation of teaching resources. Finally the authors remind us of the need to evaluate what has worked and what has not, to inform the future direction of online learning. The paper is well worth the read.

Reviewer Expertise:

NA

No decision status is available

# Reviewer report: Reviewer response for version 1

**DOI:** 10.21956/mep.20067.r30818

**Published:** 2020-04-30

**Authors:** Mike Davis

**Affiliations:** 1 freelance

This review has been migrated. The reviewer awarded 5 stars out of 5 While not a fan of “tips” for something as complex as medical education, I nevertheless find this exploration of some of the challenges facing the community to be thoughtful, thorough and helpful, in terms of identifying possible avenues to be explored in order to make the best use of resources and time.My reservations stem primarily from concerns about how the materials in all of their variety are used by learners as they engage at a distance. Pressures from a variety of sources can minimise the rigorous engagement often demanded by complex planned learning outcomes and listening to a podcast as you drive home after a demanding shift might not offer the best experience. This emphasises for me the need to maximise the use of (a)synchronous discussion opportunities that are a) thoughtfully moderated and b) challenging of understanding and application and give the opportunity, where necessary for c) sharing clinical outcomes (positive and negative) that can feed back into the shared content.This is a paper that deserves to be widely shared as medical education colleagues across the world face the COVID-19 world and continued provision beyond.

Reviewer Expertise:

NA

No decision status is available

# Reviewer report: Reviewer response for version 1

**DOI:** 10.21956/mep.20067.r30817

**Published:** 2020-04-30

**Authors:** Tharin Phenwan

**Affiliations:** 1 University of Dundee

This review has been migrated. The reviewer awarded 5 stars out of 5 This is highly relevant to everyone who needs to conduct their teaching online. The tips are extremely useful, especially tips 9 and 11.These tips wil be of interest for those who are involved in curriculum planning anyone who needs to teach online. It will be interesting to see how curriculum will change post-COVID too.

Reviewer Expertise:

NA

No decision status is available

# Reviewer report: Reviewer response for version 1

**DOI:** 10.21956/mep.20067.r30819

**Published:** 2020-04-30

**Authors:** Trevor Gibbs

**Affiliations:** 1 AMEE

This review has been migrated. The reviewer awarded 4 stars out of 5 I enjoyed reading this well written paper by the TEL Committee the Tips were very practical and applicable, I specifically liked the 'hint" that what we were going through during Covid-19 was a learning "point towards the future" moment- the world of health professions education will never return to what it previously was and the use of electronic platforms for learning are not something that only a few will use. I did feel however that this was a paper written for those already several steps up the learning curve and for those institutes relatively well-resourced. The recent AMEE webinar series regarding on-line learning showed that many global faculty colleagues are much lower down the learning curve, and are very much more resource -constrained. I was looking therefore for an element within the latter tips of evaluation of how we can be more collegial and cooperative with our lessons learned.Despite my reservations I would recommend this paper to all involved with curriculum planning during and post the Covid-19 pandemic.

Reviewer Expertise:

NA

No decision status is available

# Reviewer report: Reviewer response for version 1

**DOI:** 10.21956/mep.20067.r30816

**Published:** 2020-04-29

**Authors:** Samar Ahmed

**Affiliations:** 1 Ain Shams University Faculty of Medicine

This review has been migrated. The reviewer awarded 5 stars out of 5 This article is beyond any comments. I congratulate the excellent work and write up. I applaud the direct application if concepts if organizational change to transitioning to online learning. I hope i get to read a paper next year reflecting on application if these 12 tips post implementation. Congratulations and thank you for the amazing read

Reviewer Expertise:

NA

No decision status is available
